# Abridged version of the AWMF guideline for the medical clinical diagnostics of indoor mould exposure: S2K Guideline of the German Society of Hygiene, Environmental Medicine and Preventive Medicine (GHUP) in collaboration with the German Association of Allergists (AeDA), the German Society of Dermatology (DDG), the German Society for Allergology and Clinical Immunology (DGAKI), the German Society for Occupational and Environmental Medicine (DGAUM), the German Society for Hospital Hygiene (DGKH), the German Society for Pneumology and Respiratory Medicine (DGP), the German Mycological Society (DMykG), the Society for Pediatric Allergology and Environmental Medicine (GPA), the German Federal Association of Pediatric Pneumology (BAPP), and the Austrian Society for Medical Mycology (ÖGMM)

**DOI:** 10.1007/s40629-017-0013-3

**Published:** 2017-02-28

**Authors:** Gerhard A. Wiesmüller, Birger Heinzow, Ute Aurbach, Karl-Christian Bergmann, Albrecht Bufe, Walter Buzina, Oliver A. Cornely, Steffen Engelhart, Guido Fischer, Thomas Gabrio, Werner Heinz, Caroline E. W. Herr, Jörg Kleine-Tebbe, Ludger Klimek, Martin Köberle, Herbert Lichtnecker, Thomas Lob-Corzilius, Rolf Merget, Norbert Mülleneisen, Dennis Nowak, Uta Rabe, Monika Raulf, Hans Peter Seidl, Jens-Oliver Steiß, Regine Szewszyk, Peter Thomas, Kerttu Valtanen, Julia Hurraß

**Affiliations:** 1 Institute for Occupational Medicine and Social Medicine, University Hospital, Medical Faculty, RWTH Aachen University, Aachen, Germany; 2 Department of Infection Control and Environmental Hygiene, Cologne Health Authority, Neumarkt 15–21, 50667 Cologne, Germany; 3 Formerly: Regional Social Security Authorities (LAsD) for Schleswig-Holstein, Kiel, Germany; 4 Department of Microbiology and Mycology, Dr. Wisplinghoff Laboratory, Cologne, Germany; 5 Allergy-Centre-Charité, Charité-Universitätsmedizin, Berlin, Germany; 6 Experimental Pneumology, Ruhr University, Bochum, Germany; 7 Institute for Hygiene, Microbiology and Environmental Medicine, Medical University of Graz, Graz, Austria; 8 Department I for Internal Medicine and Cologne Excellence Cluster on Cellular Stress Responses in Aging-Associated Diseases (CECAD), University of Cologne, Cologne, Germany; 9 Institute for Hygiene and Public Health, Bonn University Hospital, Bonn, Germany; 10 Baden-Württemberg Regional Health Authorities at the Regional Council Stuttgart, Stuttgart, Germany; 11 Formerly: Baden-Württemberg Regional Health Authorities at the Regional Council in Stuttgart, Stuttgart, Germany; 12 Medical Clinic and Outpatient Clinic II with Special Focus on Infectiology, Würzburg University Hospital, Würzburg, Germany; 13 Bavarian Office for Health and Food Safety, Munich, Germany; 14 Adj. Prof. “Hygiene and Environmental Medicine”, Ludwig-Maximilian University, Munich, Germany; 15 Westend Allergy and Asthma Centre, Berlin, Germany; 16 Wiesbaden Centre for Rhinology and Allergology, Wiesbaden, Germany; 17 Clinic and Outpatient Clinic for Dermatology and Allergology am Biederstein, Technical University of Munich, Munich, Germany; 18 Medical Institute for Environmental and Occupational Medicine MIU GmbH, Erkrath, Germany; 19 Christian Children’s Hospital, Osnabrück, Germany; 20 Institute for Prevention and Occupational Medicine of the German Social Accident Insurance, Institute of the Ruhr University Bochum (IPA), Bochum, Germany; 21 Leverkusen Asthma and Allergy Centre, Leverkusen, Germany; 22 Institute and Outpatient Clinic for Occupational, Social, and Environmental Medicine, Member of the German Centre for Lung Research, Munich University Hospital, Munich, Germany; 23 Centre for Allergology and Asthma, Johanniter Hospital im Fläming Treuenbrietzen GmbH, Treuenbrietzen, Germany; 24 Formerly: Chair of Microbiology and Clinic and Outpatient Clinic for Dermatology and Allergology am Biederstein, Technical University of Munich, Munich, Germany; 25 Centre for Pediatric and Adolescent Medicine, University Hospital Gießen and Marburg GmbH, Gießen, Germany; 26 Specialist Practice for Allergology and Pediatric Pneumology, Fulda, Germany; 27 FG (specialist field) II 1.4 Microbiological Risks, Federal Environmental Agency, Berlin, Germany; 28 Department and Outpatient Clinic for Dermatology and Allergology, Ludwig-Maximilian University, Munich, Germany

**Keywords:** Mould, Health risk, Indoor, Diagnostics, Guideline

## Abstract

This article is an abridged version of the AWMF mould guideline “Medical clinical diagnostics of indoor mould exposure” presented in April 2016 by the German Society of Hygiene, Environmental Medicine and Preventive Medicine (*Gesellschaft für Hygiene, Umweltmedizin und Präventivmedizin, GHUP*), in collaboration with the above-mentioned scientific medical societies, German and Austrian societies, medical associations and experts. Indoor mould growth is a potential health risk, even if a quantitative and/or causal relationship between the occurrence of individual mould species and health problems has yet to be established. Apart from allergic bronchopulmonary aspergillosis (ABPA) and mould-caused mycoses, only sufficient evidence for an association between moisture/mould damage and the following health effects has been established: allergic respiratory disease, asthma (manifestation, progression and exacerbation), allergic rhinitis, hypersensitivity pneumonitis (extrinsic allergic alveolitis), and increased likelihood of respiratory infections/bronchitis. In this context the sensitizing potential of moulds is obviously low compared to other environmental allergens. Recent studies show a comparatively low sensitizing prevalence of 3–10% in the general population across Europe. Limited or suspected evidence for an association exist with respect to mucous membrane irritation and atopic eczema (manifestation, progression and exacerbation). Inadequate or insufficient evidence for an association exist for chronic obstructive pulmonary disease, acute idiopathic pulmonary hemorrhage in children, rheumatism/arthritis, sarcoidosis and cancer. The risk of infection posed by moulds regularly occurring indoors is low for healthy persons; most species are in risk group 1 and a few in risk group 2 (*Aspergillus fumigatus, A. flavus*) of the German Biological Agents Act (*Biostoffverordnung*). Only moulds that are potentially able to form toxins can be triggers of toxic reactions. Whether or not toxin formation occurs in individual cases is determined by environmental and growth conditions, above all the substrate. In the case of indoor moisture/mould damage, everyone can be affected by odour effects and/or mood disorders. However, this is not a health hazard. Predisposing factors for odour effects can include genetic and hormonal influences, imprinting, context and adaptation effects. Predisposing factors for mood disorders may include environmental concerns, anxiety, condition, and attribution, as well as various diseases. Risk groups to be protected particularly with regard to an infection risk are persons on immunosuppression according to the classification of the German Commission for Hospital Hygiene and Infection Prevention (*Kommission für Krankenhaushygiene und Infektionsprävention, KRINKO*) at the Robert Koch- Institute (RKI) and persons with cystic fibrosis (mucoviscidosis); with regard to an allergic risk, persons with cystic fibrosis (mucoviscidosis) and patients with bronchial asthma should be protected.

The rational diagnostics include the medical history, physical examination, and conventional allergy diagnostics including provocation tests if necessary; sometimes cellular test systems are indicated. In the case of mould infections the reader is referred to the AWMF guideline “Diagnosis and Therapy of Invasive Aspergillus Infections”. With regard to mycotoxins, there are currently no useful and validated test procedures for clinical diagnostics. From a preventive medicine standpoint it is important that indoor mould infestation in relevant dimension cannot be tolerated for precautionary reasons. With regard to evaluating the extent of damage and selecting a remedial procedure, the reader is referred to the revised version of the mould guideline issued by the German Federal Environment Agency (*Umweltbundesamt, UBA*).

## Introduction

In April 2016, the German Society of Hygiene, Environmental Medicine and Preventive Medicine (*Gesellschaft für Hygiene, Umweltmedizin und Präventivmedizin, GHUP*), in collaboration with the above-mentioned scientific medical societies, German and Austrian societies, medical associations and experts, presented the AWMF mould guideline “Medical clinical diagnostics of indoor mould exposure” [1].

This article is an abridged version of this AWMF guideline. More detailed information on all the content presented in this abridged version can be found in the AWMF mould guideline. Whenever reference is made below to the full version of the guideline, “AWMF mould guideline” is used.

The core messages of the AWMF mould guideline, which in turn contain core recommendations, are presented. The strength of recommendation is expressed using the following terms: strong recommendation: “shall”; recommendation: “should”; open recommendation: “may.”

## Core messages of the AWMF mould guideline

The problem of indoor mould exposure needs to be addressed in a more fact-based manner.

1. Relevant levels of indoor mould infestation must not be tolerated for precautionary reasons. For the assessment of damage extent, the reader is referred to the “Guideline on the prevention, investigation, evaluation and remediation of indoor mould growth” (mould guideline) issued by the German Federal Environment Agency (*Umweltbundesamt*, UBA) [2]. A revised version of the UBA mould guideline is expected in 2017.
2. The most important measures in indoor mould exposure include cause identification and appropriate remediation (see mould remediation guides [3, 5]).
3. Medically indicated indoor mould measurements are rarely helpful. In general, both quantitative and qualitative determinations of the mould species can be dispensed with in the case of visible mould infestation. Instead, identifying the cause of infestation is far more important, followed by the elimination of infestation and its primary causes.
4. Mould exposure can generally lead to mucous membrane irritation (MMI), odour effects and mood disorders.
5. Specific clinical pictures seen in mould exposure are pertinent to allergies and fungal infections (mycosis).
6. It is the physician’s duty to objectify suspected links between indoor moisture damage/mould and gastrointestinal or renal disease, reproductive disorders, teratogenicity or cancer.
7. Risk groups warranting particular protection include:
  - individuals on immunosuppression according to the classification of the German Commission for Hospital Hygiene and Infection Prevention (*Kommission für Krankenhaushygiene und Infektionsprävention*, KRINKO) at the Robert Koch-Institute (RKI) [11],
  - individuals with cystic fibrosis (mucoviscidosis),
  - individuals with bronchial asthma.
8. The risk for developing asthma (“allergic march”) is increased in:
  - patients with allergic rhinoconjunctivitis,
  - patients with allergic rhinosinusitis,
  - atopic patients.
9. It is likely that all moulds are capable of causing sensitization and allergies. Their allergenic potential is considered lower compared with other environmental allergens [12, 13].
10. As polysensitized individuals, atopics (those susceptible to hypersensitivity reactions, such as allergic rhinitis (hay fever), allergic asthma, and atopic dermatitis on contact with environmental substances) often also exhibit IgE antibodies to moulds; however, this does not necessarily indicate relevant disease.
11. The core elements of allergy diagnostics include medical history, skin testing (skin prick test), *in vitro* serological examination of specific IgE antibodies in type I sensitization or specific IgG antibodies in hypersensitivity pneumonitis (HP; extremely rare in non-occupationally related indoor exposure) and provocation testing.
12. The identification of specific IgE means that a specific sensitization to relevant allergens is present. However, this cannot be equated to a clinically relevant allergy any more than a positive skin test reaction can be.
13. Negative *in vitro* and *in vivo* tests do not exclude sensitization or mould allergy.
14. The determination of specific IgG antibodies as part of the diagnostic work-up for immediate-type mould allergy (type I allergy) is of no diagnostic relevance and is therefore not recommended.
15. Lymphocyte transformation testing (LTT) for moulds is not indicated as a diagnostic method [14].
16. Mould-related infections are rare and are most likely to occur via the inhalative route. In practice, *Aspergillus fumigatus*—the most important mycotic pathogen—is of the greatest relevance among the 460 moulds classified in risk groups 2 and 3 according the German Technical Rules for Biological Materials (*Technische Regeln für Biologische Arbeitsstoffe*, TRBA). Individuals with local or general immunodeficiency are by far those most frequently affected.
17. Core elements of the diagnostic work-up for mould infection include microbiological, immunological, molecular biological and radiological methods.
18. Mould-allergic individuals, as well as patients with diseases that weaken the immune system, should be provided with specialist patient information on the hazards of indoor mould exposure and the preventive steps that can be taken to minimize this exposure.

## Aim of the AWMF mould guideline

The guideline is intended to close the existing gap in the rational and efficient medical diagnostics of indoor mould exposure. To date, only guidelines on building-related procedures in the case of moisture damage [2–6] and overview articles on associated diseases [7–10] have been available—however, no comprehensive, patient-related diagnostic procedure.

The AWMF guideline does not address workplace-related diseases or specific workplace exposure, oral ingestion of moulds or mould components or yeast- and dermatophyte-related diseases.

The scientific literature on moulds is extensive and largely published in English. In epidemiological studies, domestic exposure is often categorized using the terms “dampness and mould,” i. e. no distinction is made between indoor moisture damage with or without mould infestation. This makes sense, since there is no health-related marker for quantitative mould exposure. “Mould” (or “mold” in American English) refers to “visible” mould structures, whereby “visible” also includes hidden mould infestation. The terms “dampness and mould” are translated in the AWMF mould guideline as “*Feuchtigkeit und Schimmel*”. Further definitions can be found in the appendix to the AWMF mould guideline [1].

## Methodology

A national network of experts belonging to the German Society for Hygiene, Environmental Medicine and Preventive Medicine (*Gesellschaft für Hygiene, Umweltmedizin und Präventivmedizin*, GHUP) was used to compile the AWMF mould guideline. The guideline builds on the statements of the Robert Koch-Institute (RKI) Commission ‘Methods and Quality Assurance in Environmental Medicine’ (*Kommission Methoden und Qualitätssicherung in der Umweltmedizin*) [8], the World Health Organization (WHO) Guidelines for Indoor Air Quality: Dampness and Mould [9] and the scientific workshops held at the GHUP annual conferences (GHUP 2009–2012) on the subject of “mould and health” [15–18].

The present guideline has been drawn up in accordance with the methodological requirements for the development of diagnostics and treatment guidelines set out by the German Association of Scientific Medical Societies (*Arbeitsgemeinschaft der wissenschaftlichen medizinischen Fachgesellschaften*, AWMF) and represents an S2k guideline according to the AWMF three-level concept. The guideline is based on an extensive and systematic literature search; however, it does not formally meet the requirements for an S2k guideline, since there are no clinical studies available on this topic. Thus, it was not possible to assign evidence levels to recommendations.

A search in the Cochrane database with the terms “mold”, “mould” and/or “dampness” produced three hits. Two reviews discussed specific immunotherapy in asthma and rhinitis, while one review analyzed the preventive effect on respiratory diseases of remediating damage caused by dampness and mould [19].

A conceptually graduated Medline search yielded 1949 references; screening was subsequently performed for limitation purposes. No reviews comprehensively or extensively addressing the topic of medical diagnostics in exposure to indoor mould and dampness were found, but rather only literature on individual topic areas. More details on these can be found in the AWMF mould guideline [1].

The search was conducted in German using Internet search machines (Google) and in English in the Medline (Medical Literature Analysis and Retrieval System Online) database.

Publications by the WHO [9], the Institute of Medicine (IOM, USA) [7], Palatya and Shum [20] as well as a recently published review by Mendell et al. [21] were used to make basic evaluations of the evidence of a link between mould exposure and defined clinical pictures.

Other guidelines were considered for individual topics, primarily diagnostics. These are listed in the relevant chapters of the AWMF mould guideline [1]. Other guidelines that were consulted include guidelines on the following: inhalant mould exposure; allergic rhinoconjunctivitis; rhinitis; rhinosinusitis; diagnostics and treatment of patients with asthma and bronchial asthma in children and adolescents; asthma treatment; urticaria; diagnosis of HP; allergic bronchopulmonary aspergillosis (ABPA) in cystic fibrosis; diagnostics and treatment of invasive *Aspergillus* infections; treatment of invasive aspergillosis; diagnostics and treatment of invasive fungal infections; sarcoidosis in childhood and adolescence; management of early rheumatoid arthritis; diagnostics and treatment of adult patients with acute and chronic cough; *in vitro* allergy diagnostics; skin testing to diagnose immediate-type allergic reactions; the performance of nasal provocation tests; the performance of bronchial provocation tests; workplace-specific inhalation tests; (allergen-)specific immunotherapy in IgE-mediated allergic disease; and allergy prevention (see the AWMF mould guideline for a detailed list [1]).

The procedure used to draw up the guideline is presented in detail in the AWMF mould guideline [1].

## Incidence, exposure and health relevance of moulds

### Definition and increased incidence of moulds

**Mould** is a collective term for hyphae- and generally also spore-forming micro-fungi and does not represent a taxonomically defined fungal entity.

Moulds are a ubiquitous component of our biosphere and are found to varying degrees in outdoor air, in indoor areas, and in some workplaces.

**Mould infestation** (of materials) is considered to be present in building materials and fixtures that have been, or still are, covered (colonized) by mould. Unless already visible to the naked eye, determination is performed by means of microscopic identification of a network of hyphae and, by and large, fully developed conidia- or sporangia-bearers, irrespective of whether the moulds are still vital/active or have already died off. Other biomaterial, e. g. bacteria, may also be present besides moulds.

**Mould contamination** exceeds the general background levels of contamination of surfaces or materials (e. g. with fungal spores) via entry from outside (e. g. in house dust, airborne spores).

**Mould growth** refers to a process involving biological activity, i. e. it is associated with moisture and characterized by cell division, hyphae, mycelium and potentially spore formation, among other things.

**Moisture damage** is the visible, measurable or perceived effect of increased water content in indoor areas or structural components.

It makes sense from a practical point of view to summarize increased indoor exposure to moulds and other factors associated with increased moisture, such as yeasts, bacteria (Actinobacteria) and mites, as moisture/mould damage.

### Classification of moulds

Fungi are eukaryotes with cell walls consisting of chitin and glucans.

The nomenclature of fungi is binomial, i. e. each organism bears the name of a genus and a species. However, renaming is relatively common in fungi due to continuous new findings and taxonomic classifications. This can lead to communication problems, e. g. when medical experts specify mould species found indoors (named differently in the new nomenclature in the meantime) in their report and include possible health-related problems in their appraisal. The MycoBank, an online database, provides current names, combinations and associated data, e. g. descriptions and illustrations (http://www.mycobank.org/).

In medical mycology, on the other hand, fungi are clinically classified into dermatophytes, yeasts and moulds, irrespective of taxonomy. Although the DHS system represents a practicable classification, it is misleading and, from a biological perspective, (taxonomically) incorrect, since moulds are not a taxonomical entity and most “yeasts” (yeast-like fungi) belong taxonomically to the *Ascomycota*, as do dermatophytes.

From a microbiological perspective, moulds should generally be given taxonomically as genus and species. If only the Latin genus name is given followed by sp. or spp., the particular species or group of species have not been further differentiated.

Another classification with a practical focus is made according to the different temperature and moisture requirements of the individual moulds [22, 23].

### Mycotoxins

Mycotoxins are secondary metabolites produced by moulds, which, in low concentrations (µg/kg foods), can have toxic effects on various cell systems in vertebrates irrespective of the type of toxin and consumption habits. Numerous mould genera (e. g. *Aspergillus, Penicillium, Fusarium, Alternaria, Stachybotrys*) produce mycotoxins. Mycotoxin production depends on the species and environmental factors, such as substrate composition, moisture levels, pH value, light wavelength and nutrient competition [24]. As a general rule, mycotoxins produced by indoor-relevant moulds can be detected in extremely low concentrations (parts per trillion, ppt) in house dust [25], bioaerosols and building materials. Mycotoxins are not volatile and are found in the air bound to spores, cell fragments and other particles.

They are generally only found at levels relevant to health in foods and animal feed that have been colonized by mould.

The hitherto available data indicate that the levels of most airborne mycotoxins found indoors do not exhibit an acute toxic effect. Only the strongest toxic compounds, trichothecenes and gliotoxins, may be found at their effective concentrations as a result of mould-infested material indoors [26].

Clearly, the maximum expected levels of individual mycotoxins *in situ* (bioaerosols) do not alone explain these cytotoxic effects. It would appear that the synergistic effects of various mycotoxins, or mycotoxins with different cell components (e. g. glucanes, endotoxins), are more likely to be responsible for this effect [8].

It is not yet possible to rule out the possibility that airborne concentrations of the compounds gliotoxin in *Aspergillus fumigatus* (only rarely relevant indoors) and the satratoxins in *Stachybotrys chartarum *reach a level that may be responsible for immunomodulatory effects and, thus, potentially promote susceptibility to infection or allergy development [27].

### Cell wall components and metabolites

Besides mould spores and mycotoxins, other metabolites and cellular components, such as microbiological volatile organic compounds (MVOC), β‑glucans, mannans and ergosterol, also play a role in mould exposure [28, 29], whereby MVOC are responsible for the characteristic odour of mould.

Ergosterol is a metabolite (sterol) of yeasts, moulds and edible mushrooms. It is produced as a membrane component in varying quantities.

Moisture damage is also accompanied by other microbiological components (e. g. found in house dust), such as the lysosomal enzyme N‑acetyl-ß-D-glucosaminidase and endotoxin (in bacteria) [30]. It is not yet known whether these markers (cell fragments, ß‑glucan and ergosterol) correlate better with health effects than do culturable exposure parameters [31–37].

To date, 77 mould allergens (excluding dermatophytes and yeasts) have been described and officially recognized (www.allergen.org). The associated protein families differ significantly both biochemically and structurally from the allergen families in pollen, foods and animal dander [38].

### Health problems and diseases caused by moulds

Epidemiological studies show, consistently and across studies, a relationship between indoor moisture damage and health effects, in particular: respiratory symptoms; eye, nose and throat irritation; blocked nose; wheezing; dry cough; and fatigue [39]. The AWMF mould guideline confines itself largely to clinical pictures rather than symptoms.

The relevant evidence of links between moisture/mould damage and its various health effects is summarized in Table 1. In some cases, it is not possible to unequivocally establish causality between specific mould exposure and concrete health-related problems and clinical pictures.

**Table 1:** Evidence of a link between indoor mould exposure or dampness and disorders (excluding mycoses) (modified from [9, 20, 21, 40, 41])

**Causal link** Insufficient evidence
**Sufficient evidence for an association:** Allergic airway diseases Asthma (manifestation, progression, exacerbation) Allergic rhinitis Hypersensitivity pneumonitis (extrinsic allergic alveolitis) Promotion of airway infections, bronchitis
**Limited or suspected evidence for an association:** Mucous membrane irritation (MMI) Atopic eczema (manifestation, progression, exacerbation)
**Inadequate or insufficient evidence for an association:** Chronic obstructive pulmonary disease (COPD) Acute idiopathic pulmonary haemorrhage in infants Rheumatic disorders, arthritis Sarcoidosis Cancer

Whether moulds pose a health risk largely depends on the disposition of the exposed individuals. Risk groups warranting particular protection include:

- individuals on immunosuppression according to the Commission for Hospital Hygiene and Infection Prevention (*Kommission für Krankenhaushygiene und Infektionsprävention*, KRINKO) [11],
- individuals with cystic fibrosis (mucoviscidosis),
- individuals with bronchial asthma.

Ensuring that living conditions meet the highest possible standards of hygiene should be a fundamental requirement in all chronic diseases, including those with no, or insufficient, evidence of a link to moisture damage and/or mould exposure. If hygiene conditions or medical history suggest moisture damage and/or mould exposure, the primary causes need to be preventively eliminated, as with all moisture damage [42].

### Defined clinical pictures and health disorders

No single mechanism or factor is able to explain the various health effects related to moisture damage and/or mould exposure [21, 43, 44]. Epidemiological findings point to both allergological and non-IgE-mediated immunological and toxic, immunomodulatory mechanisms. Moisture damage or mould growth can cause adverse effects in atopic as well as non-atopic individuals [45–48].

The sequence in which diseases are presented in the following is not intended to indicate any order of priority in relation to the topics addressed in the AWMF mould guideline.

#### 1. Allergic rhinitis

As polysensitized individuals, atopics (i. e. persons with allergic asthma, allergic rhinitis, atopic dermatitis) often exhibit IgE antibodies against moulds.

Depending on the population, region and allergen spectrum investigated, the incidence of allergic rhinitis due to fungal allergens is given at rates ranging from 2.7–19% [49–51].

IgE-mediated rhinitis is most commonly elicited by allergens in moulds predominantly found in ambient air, in particular *Alternaria alternata*, and significantly less frequently *Cladosporium herbarum, Botrytis cinerea, Mucor *sp., *Penicillium *sp. and *Aspergillus *sp. [49–51]. In epidemiological studies, indoor dampness and mould are consistently associated with allergic rhinitis [21, 52]. Monosensitization to indoor moulds, however, is likely to be rare [53].

#### 2. Non-invasive and invasive sinusitis

Moulds can trigger chronic inflammation of the nasal and paranasal sinus mucosa via a variety of mechanisms [54, 55]. Among sensitizations to moulds in patients with chronic sinusitis, *Alternaria*, a typical mould in outdoor air, is the most prevalent [56].

A distinction is currently made between five forms of rhinosinusitis triggered by fungi:

- acute invasive (including rhinocerebral mucormycosis),
- chronic invasive,
- granulomatous invasive,
- non-invasive allergic fungal rhinosinusitis (AFRS) without and
- with spherical mycetoma formation [54, 57].

The invasive forms are more prevalent in immunocompromised patients (AIDS, diabetes, chemotherapy etc.) and can cause death within a matter of weeks in the case of an acute (fulminant) course involving vascular invasion by hyphae. The chronic invasive form, on the other hand, follows a protracted course and, here again, immunosuppressed patients are predominantly affected. The granulomatous invasive form represents a type of fibrotic tumour formation, occurring primarily in Africa, Saudi Arabia and the Arab Gulf States.

Non-invasive AFRS was first described in conjunction with ABPA [58]. AFRS also resembles this bronchial disease in many respects. Dematiaceous hyphae (*Bipolaris spicifera, Curvularia lunata*) and *Aspergillus* species (e. g., *Aspergillus fumigatus, A. niger* and *A. flavus*) are most commonly found to be the trigger [59].

The presence of a thick, tenacious secretion and the typical histological finding of abundant eosinophils is clinically characteristic [60]. In the US, the diagnosis is considered confirmed if all the major criteria of the Bent & Kuhn classification are met:

1. Type 1 allergy to fungal allergens confirmed by skin testing or *in vitro* testing.
2. Nasal polyposis.
3. Characteristic computed tomography findings.
4. The presence of eosinophilic mucin without invasion.
5. Positive fungal stain of sinus contents removed at surgery [60, 61].

Recent studies have shown that fungi can be found in the nose and paranasal sinuses of the vast majority of the population (including all CRS patients) [62]. Thus, alone the presence of fungi does not appear to be pathognomic—and hence diagnostically significant—but instead an expression of (a) a reduced immune response in invasive fungal diseases or (b) an altered, partially excessive immune response in AFRS to these ubiquitously occurring fungal spores.

Therefore, from a therapeutic perspective, treatment with topical and oral antifungal agents is only recommended in invasive forms, not however in AFRS, since double-blind placebo-controlled studies have not been able to show an effect for these agents [62], and a pathophysiological relationship to moulds cannot be assumed in the majority of CRS cases [63].

According to recent findings, severe, untreatable CRS is caused by (fungal, among other) biofilms. The precise pathomechanism has not yet been elucidated. It is likely that planktonic fungi are continually released by the biofilm; as part of this process, the mucosa is probably invaded by macrophages that phagocytose—but do not kill off—the fungal hyphae [64–67].

Fungal biofilms are made up of micro-fungal complexes that are capable of colonizing both biotic and abiotic surfaces. They cause circumvention of the immune system and reduce sensitivity to antifungal agents, while maintaining the ability to release planktonic micro-fungal hyphae. Numerous investigations using different detection methods have been able to demonstrate the presence of biofilms in the sinonasal mucosa of CRS patients [64–67]. The presence of biofilms was associated with poorer disease courses [64]. In patients requiring surgery, preoperative disease severity was greater in a patient group with proven biofilms in the sinonasal mucosa compared with a control group in whom no relevant biofilms were detected; however, the postoperative outcome was identical in both groups [66].

Confocal scanning laser microscopy with fluorescent *in situ* hybridization was deemed the “gold standard” in terms of biofilm detection methods [67]. This method should be combined with other microbiological investigations. Traditional culture techniques to detect and identify pathogens complement this diagnostic work-up [67]. Thus, biofilms are an interesting approach to explaining the persistence of moulds in the chronically inflamed sinus mucosa. The clinical significance of biofilms to disease course cannot be fully assessed as yet. It would be important in the future to develop suitable detection methods for routine clinical application.

#### 3. Allergic bronchial asthma

As in allergic rhinitis, seasonal allergic bronchial asthma is primarily induced by moulds occurring at seasonally high levels in outdoor air (e. g. generally *Alternaria*, more rarely *Cladosporium, Epicoccum, Fusarium*), whereas indoor moulds (*Aspergillus, Penicillium*) cause perennial allergic bronchial asthma [8, 68]. The link between damp indoor environments and/or mould and the development of asthma, particularly in children, can be considered as undisputed [21, 69–72].

The outdoor mould genus, *Alternaria alternata *(formerly *A. tenuis*), appears to be a particularly important mould in the context of asthma development and severity [73–77]. A temporal relationship between asthma symptoms and spore counts was seen particularly in cases of a high degree of sensitization, as well as in patients without concomitant grass pollen allergy. Other authors have emphasized the relevance in allergic asthma of *Cladosporium* sp., extremely high levels of which are found seasonally in outdoor air, as well as in cases of indoor infestation [78–83]. In rare cases, patients with seasonal asthma symptoms (June to September) may exhibit *Alternaria* sensitization without concomitant pollen sensitization [84].

Allergic bronchial asthma is often accompanied by other atopic diseases (atopic dermatitis, allergic rhinoconjunctivitis) [8, 85–89]. Monosensitization to indoor moulds is rare. Clinical investigations show that, in the case of mould, polyvalent sensitizations to other environmental allergens are often present [90]. Iversen and Dahl [91] also provide evidence that up to 95% of mould-allergic asthmatics were additionally sensitized to other inhalant allergens. The authors conclude that mould allergens, as weak allergens, only rarely induce monovalent allergies, or these generally only occur in patients with a high sensitization potential, and that genetic predisposition is more relevant in this sensitization process than is mould exposure in damp homes [91, 92].

#### 4. Atopic dermatitis (atopic eczema)

As airborne allergens, mould allergens can likely trigger atopic dermatitis [86–88]. Epidemiological studies have yielded sufficient evidence to support a link between atopic dermatitis and moisture damage/mould [21].

A variety of dermatological reactions to mould have been described, such as dryness, pruritus and skin rashes [93, 94]. Whether this represents an immunologically mediated form of skin reaction to indoor mould exposure is unclear [44]. However, occupational contact dermatitis in conjunction with mould exposure can also be a manifestation of immunologically mediated dermatitis in mould sensitization [95].

#### 5. Urticaria

In rare cases, the ingestion of foods contaminated by mould components can trigger urticaria [86, 87]. Examples include mould components (such as enzymes) in beverages and bakery products or on dry fermented sausage/salami [96–98]. Airborne exposure as trigger of urticaria is unlikely [20] or extremely rare [99]. Occupational contact urticaria in conjunction with mould exposure may also be a manifestation of immunologically mediated dermatitis in mould sensitization [97].

#### 6. Hypersensitivity pneumonitis (HP)

Clinical evidence has documented a link between HP (synonym: extrinsic allergic alveolitis [EAA]) in susceptible individuals and the occurrence of mould [7]. With a prevalence of between two and four cases per 100,000 inhabitants/year, HP is a rare allergy (type III, IV) to inhalant antigens [100, 101]. Indoor mould plays an important role in this rare disease. The antigens are found in dust and aerosols; possible microbially contaminated sources include, for instance, birds, feathers, hay, wood dust, air humidifiers, air-conditioning systems, indoor fountains, aquariums and steam irons [102–104]. Most commonly, the antigens come from birds, moulds and actinomycetes [105]. Non-smokers are predominantly affected by HP. Sennekamp [106] has put together a comprehensive antigen catalogue. HP occurs primarily in the workplace [107] and belongs to the recognized occupational diseases (OD No. 4201). Non-workplace-related cases are extremely rare [108–111]. In central Europe, bird-fancier’s lung is the predominant clinical picture [106, 112–114].

#### 7. Allergic bronchopulmonary aspergillosis (ABPA)

Allergic bronchopulmonary aspergillosis (ABPA) is a rare immunological lung disease involving sensitization (IgE and IgG antibodies) to *Aspergillus* antigens. It is caused by the inhalation of, and colonization by, *Aspergillus* spores that trigger an immune reaction. As part of this process, fungi may grow in the mucus—but not in tissue—and form hyphae. More rarely, allergic bronchopulmonary mycoses caused by *Helminthosporium, Candida* or other fungi may induce a similar picture. The clinical presentation of ABPA includes cough, worsening asthma, hemoptysis and tenacious mucus leading to mucus plugging. ABPA should be considered if more than two of the following criteria are met: cystic fibrosis; bronchial asthma; eosinophilia of unknown etiology; volatile antibiotic-resistant infiltrates; acquired central bronchiectasis; *Aspergillus* detection in sputum; expectoration of brownish mucus plugs; delayed cutaneous reaction to *Aspergillus*. The diagnosis of ABPA is based on the modified criteria originally proposed by Rosenberg et al. ([115]; Table 2). Recent investigations show that the combination of elevated total IgE (>1000 IU/l) and specific IgE against rAsp f 4 and rAsp f 6 permits the diagnosis of classic ABPA to be made with 100% specificity and 64% sensitivity. Treatment consists primarily of oral steroids; it is still not possible to generally recommend antifungal treatment. There are initial indications that treatment with omalizumab is able to reduce steroid requirements. Since ABPA can cause progressive fibrotic lung changes if left untreated, early diagnosis and treatment are important [116–120].

**Table 2:** Diagnosis criteria for allergic bronchopulmonary aspergillosis (*ABPA*) [117, 118, 120] (modified from Rosenberg et al. [115])

Diagnostic criteria for allergic bronchopulmonary aspergillosis (ABPA)	If not all criteria are met, ABPA is likely in the presence of the following minimal criteria:
Specific IgG antibodies (precipitins) against *Aspergillus* sp	Asthma
Specific IgE antibodies against *Aspergillus* sp	Immediate-type cutaneous reaction to *Aspergillus* sp
Total IgE (>1000 kU/l)	Transient pulmonary infiltrates
Detection of rAsp f 4 and rAsp f 6	Elevated total IgE
Recurrent asthma	Specific IgG and IgE antibodies against *Aspergillus fumigatus*
Recurrent transient pulmonary infiltrates	–
Immediate-type cutaneous reaction to *Aspergillus* sp	–
Blood eosinophilia, possibly sputum eosinophilia	–
Central bronchiectasis	–

#### 8. Mycoses

Infections caused by environmental fungi are referred to as exogenous mycoses. The diagnostic work-up and treatment of mycoses do not form part of the AWMF mould guideline; instead, only an assessment is made of the risk of infection upon exposure to indoor mould, since at-risk patients require individualized medical advice regarding consequences and preventive measures.

Fungal infections have increased in recent years [121, 122]. High incidence rates are seen above all in hemato-oncological patients with long phases of neutropenia, as well as in recipients of allogeneic stem cell transplantation. However, other forms of immunosuppression, such as prolonged corticosteroid use and interstitial lung disease (including residual cavitation, e. g. following tuberculosis [123, 124]), as well as a combination of these factors, especially in chronic obstructive pulmonary disease (COPD), have been linked to increased mould infection rates [125, 126]. Thanks to improved treatment options, hematological and oncological patients can now be treated for longer periods. However, this often leads to longer periods of increased infection risk, as well as recurrent neutropenia. Moreover, there is a trend toward relocating inpatient chemotherapy to the home environment [127]. This can result in increased exposure in the domestic setting during and/or directly after chemotherapy. Mould infections are among the most frequent causes of death due to infectious disease in hemato-oncological patients, and they are gaining in significance [121]. Mould-related mycoses in susceptible patients are usually acquired via the airways. Primary sites of infection are most commonly the lung and more rarely the paranasal sinuses, ear or injured skin. Originating in the respiratory tract, moulds can spread hematogenously or lymphogenically, thereby affecting other organs [23].

Although heat-tolerant *Aspergillus* species are only rarely found at high levels indoors (potentially in plant pots), they can be carried into indoor areas, e. g. due to close proximity to compost or waste treatment facilities, or as a result of anthropogenic effects (e. g. agricultural activities).

Individual cases of infection due to opportunistic moulds (mesophilic “environmental” species) have been described in the literature [128–133]. A recent analysis of altogether 53 aspergillosis outbreaks affecting 458 patients identified *Aspergillus fumigatus* and *Aspergillus flavus* as the most common species. Over 50% of affected patients came from hematology/oncology departments.

In the hospital setting, (nosocomial) mould infections occur primarily as a result of *Aspergillus* and *Mucor* spore inhalation, contaminated materials, construction work or potted plants. Nosocomial infections are defined as the diagnosis of an infection >48 h following inpatient admission. Immunosuppression generally occurs later, following chemotherapy lasting for several days. Spore inhalation, on the other hand, can occur earlier and also prior to inpatient admission. In this way, spores on the mucosa (e. g. the paranasal sinuses) may persist and only cause infection upon immunosuppression. This likely explains infections that occur even in maximum isolation and with high-efficiency particulate arrestance (HEPA) air filtration. As case studies demonstrate, mould infections can also occur outside the hospital setting [134–138]. The investigations conducted by Chen et al. [135] in Taiwan on pulmonary fungal infections revealed an increase in community acquired fungal infections. The link between building sites and demolition works and the resulting increase in fungal spore exposure in outdoor, as well as (secondarily) indoor, air is considered as established [139].

With regard to all reports on fungal infections, it must be borne in mind that it is not unequivocally clear whether these infections were acquired outside the hospital setting and/or outside of indoor spaces.

##### 8.1 Invasive aspergillosis.

Invasive *Aspergillus* infections are an important cause of morbidity and mortality in immunodeficient patients [140, 141]. Insufficient data is available on the incidence of aspergillosis in Germany; it is associated with high mortality rates (30–95%) in the over 200,000 annual cases of life-threatening *Aspergillus* infection worldwide [142].

The reader is referred to the joint guideline currently being drawn up by the German-speaking Mycological Society (*Deutschsprachige Mykologische Gesellschaft*, DMYKG) and the Paul Ehrlich Society (*Paul-Ehrlich-Gesellschaft*, PEG) for the diagnosis and management of (angio-)invasive bronchopulmonary aspergillosis, as well as to the “Invasive Fungal Infection” guideline according to the German Specialist society for the Diagnostics and Treatment of hematological and oncological Diseases.

##### 8.2 Aspergilloma.

Aspergilloma (mycetoma or fungus ball) is a localized form of aspergillosis that generally develops in preformed cavities (paranasal sinus, lungs) due to a build-up of mould mycelia. Predisposing factors include, e. g. caverns secondary to tuberculosis, bronchiectasis and malignant disease [143, 144].

#### 9. Organic dust toxic syndrome (ODTS)

Organic dust toxic syndrome is an acute, systemic flu-like disease caused by the inhalation of high concentrations of bioaerosols found almost exclusively in workplaces. It is significantly more common than HP (see Section “Toxicological diagnostics”), from which it is sometimes difficult to distinguish diagnostically [23, 102, 145]. Table 3 provides a decision-making aid for the differential diagnosis between HP versus ODTS. ODTS symptoms have been described in extremely high bioaerosol exposure. Exposure to high quantities of dust with an extreme bacterial load (>10^9^ spores/cubic meter, possibly less for *Aspergillus fumigatus*) [146] can cause asthma and pneumonitis [147], resembling HP symptoms. Granulomatous scarring and pulmonary fibrosis may be seen in the case of continued exposure [146, 148]. Details on the precise cause of the toxic irritant effect in ODTS are not known [4, 8].

**Table 3:** Differential diagnosis between hypersensitivity pneumonitis (*HP*) and organic dust toxic syndrome (*ODTS*) [8]

Characteristics	HP	ODTS
Exposure	Various allergens	Endotoxins, high exposure
Incidence	2–30/10,000	10–100/10,000
Latency	4–8 h	4–12 h
Auscultation	Bilateral basal end-expiratory crackles	Normal, crackles possible
Pulmonary function	Restricted (infrequently obstructed, low D_LCO_)	Normal (possibly restricted)
Precipitins	Often specific IgG	Usually negative

#### 10. Pulmonary hemorrhage and acute idiopathic pulmonary hemosiderosis (AIPH)

There is no rationale at present to assume a causal link between pulmonary hemorrhage and the presence of indoor mould [149, 150]. Nevertheless, a degree of association cannot be ruled out [151]. Medical history taking in children with AIPH should include questions about dampness/mould [152].

#### 11. Susceptibility to infection

There is evidence of a consistent association between water damage or indoor mould exposure and the development of medically diagnosed respiratory tract diseases (common cold, bronchitis, infections) [21].

Fisk et al. [41] estimate that 8–20% of respiratory tract infections in the US are associated with mould or indoor dampness. The link continues to exist even after controlling for independent variables. *Penicillium* sp., *Cladosporium* sp., zygomycetes and *Alternaria* sp. proved to be most closely linked to the development of these diseases.

The mechanism of this association appears to be non-allergic in nature [153].

#### 12. Irritant effects—mucous membrane irritation (MMI) and chronic bronchitis

Besides a variety of environmental factors, dampness [154] and moulds [155] are associated with mucosal irritation, referred to as mucous membrane irritation (MMI)^1^, and chronic bronchitis [156]. Although the pathophysiological links between exposure to these environmental factors and MMI or chronic bronchitis have not been elucidated as yet, the mucosal epithelium and local neurons have been attributed with a key role in MMI [157]. According to a Danish study, long-term exposure to damp indoor spaces causes mucosal hyperreactivity in nasal histamine provocation that persists even after remediation [158].

The prevalence of mucosal irritation among individuals occupationally or environmentally exposed to bioaerosols is put at approximately 20–30% [159–161]. There are no reliable data as yet—in general or specifically for indoor mould exposure—on the prevalence of these non-allergic, irritant, inflammatory effects.

Possible irritant symptoms in MMI include nonspecific irritation of the mucous membrane of the eye (e. g. burning, watering, itching), the nose (e. g. sneezing, secretion and obstruction of the nasal cavity) and the throat (e. g. feeling of dryness, clearing of the throat). In addition, irritant inflammatory processes in the deeper airways (e. g. cough) may manifest as chronic bronchitis [156]. Symptoms seen during exposure, such as coughing, burning, itching of the eyes and nose and skin irritation, resolve rapidly once exposure ceases. From a differential diagnostic perspective, it is important to distinguish allergic symptoms that, unlike irritant reactions, generally increase upon repeated and long-term exposure due to sensitization [162]. The irritant toxic effects of moulds can possibly be attributed to metabolites or cell wall components (glucans), as well as to a reaction to the release of interleukins or other inflammatory mediators [39]. As part of this, synergistic effects of various mycotoxins and/or mycotoxins with other microbiological agents (e. g. glucans, endotoxins from bacteria) may be responsible for this effect [32, 34, 163–165].

#### 13. Sarcoidosis and moulds

In sum, there is only unreliable evidence that different forms of microbial inhalation exposure, including water damage, can increase the risk of developing sarcoidosis; no causal link between mould exposure and sarcoidosis has been established as yet [166, 167].

Thus, in future studies on the etiology of sarcoidosis, it would make sense to pose questions regarding inhalant—including infectiological—factors and water damage in patients’ domestic and occupational environments during medical history taking [168–173]. However, there is currently insufficient data to assume a causal link between the development or exacerbation of sarcoidosis and water damage or mould exposure.

No specific mould-related diagnostic work-up is indicated in sarcoidosis above and beyond the usual procedure.

#### 14. Rheumatic disorders

Infections (bacterial, viral) have long been discussed as triggering factors in numerous inflammatory rheumatic diseases. A working group has produced evidence of a link between moisture damage and rheumatic disorders [174–177]. The occurrence of a cluster in one building was attributed to the presence of moisture damage and “abnormal” microbial exposure [175].

However, until studies from other centres (and countries) are available, one cannot assume that the current evidence is sufficiently robust. Given that the epidemiological evidence is insufficient, it is not possible to make any statements on incidence or any possible links between mould exposure and/or moisture and rheumatic disease.

No specific mould-related diagnostic work-up is indicated in rheumatic disorders (interdisciplinary guideline, management of early rheumatoid arthritis) above and beyond the usual procedure.

#### 15. Mycotoxicosis

Systemic effects (poisoning) caused due to the mycotoxins produced by moulds are referred to as mycotoxicosis and are known to occur upon oral ingestion of contaminated foods [105].

There is no reliable knowledge to date of indoor airborne mycotoxin poisoning. It also remains to be established whether mycotoxin levels in indoor air are relevant in terms of a systemic toxicological risk. According to the findings available to date, this does not appear to be the case.

#### 16. Odour effects

Mould metabolites can cause relevant odours to be perceived [178]. This should prompt a structural investigation into the cause of indoor moisture/mould damage.

The term microbial volatile organic compounds (MVOC) refers to volatile organic compounds produced by moulds and bacteria [178–180]. It is important to bear in mind that there are also other sources of MVOC besides microbial sources (tobacco smoke, cooking, baking, roasting, pot plant soil, compost bin etc.) [181]. It has not yet been elucidated whether biological signalling effects come from MVOC at the low µg/m^3^-range levels found indoors [182]. Olfactory–psychological coupling reactions with nonspecific symptoms are possible in the case of cacosmia-related abnormalities; toxic reactions, on the other hand, are unlikely [183, 184].

Environmental odours can affect health and well-being in various ways. A distinction needs to be made between: direct physiological effects; odour perception; odour pollution as an effect of the odour on an emotional level; and indirect physiological effects due to odour pollution and the resulting chronic stress. In the reality of environmental health analysis, it is not always possible to distinguish between the health effects caused by odours via the above-mentioned mechanisms.

The characteristic effect of unpleasant odours is that they pose a nuisance. Although mood disorders as a health effect are possible, these are not mediated via toxicological mechanisms, but rather via conditioning, attribution (of links), or stress. Mood disorders can be understood as precursors to somatic dysfunction. Typical symptoms due to highly unpleasant odour pollution can include fatigue, lack of concentration, nausea, headache and insomnia [185].

The perception and cognitive appraisal of—and thus also sensitivity to—odours are subject to considerable inter-individual variability. Genetic and hormonal factors, as well as character, context and adaptation effects, play a role here [186].

#### 17. Mood disorders and nonspecific symptoms

Mood disorders are defined as “a deterioration in psychological, physical, and social well-being and feeling of subjective performance capacity. As an emotional experience, they need to be distinguished from stress responses, which include a cognitive evaluation of specific environmental stimuli” [187, 188]. Mood disorders play a crucial role in environmental health disorders in general, as well as in health disorders related to indoor environments in particular [189]. Three models are used to explain the mode of action of these types of environment-related mood disorders: the noxious agent model, the attribution model and the stress model [187, 188]. It is possible in principle that environment-related mood disorders could be triggered by moulds, e. g. as a result of odours [189].

#### 18. Neuropsychological and neurotoxic effects

The specialist literature does not point to a consistent causal relationship between indoor toxin levels and neurotoxic effects [43, 190–193]. Evidence of a link is insufficient [194].

#### 19. Gastrointestinal effects, renal effects, teratogenicity and cancer

To date, there have been no systematic investigations or case descriptions that provide evidence of, or suggest an association between, indoor moisture damage or mould and gastrointestinal or renal disease, reproductive disorders, teratogenicity or cancer (see [20, 150]).

It is the physician’s duty to investigate a possible causal link in such cases.

## Risk analysis and assessment

### Risk of infection

The risk of infection from common indoor mould species is low in healthy individuals; most species are classified in risk group 1 and only a handful in risk group 2 (*Aspergillus fumigatus, A. flavus*) of the German Biological Agents Act [195].

The current German Biological Agents Act, which regulates occupational tasks involving (the handling of) moulds, classifies the risk of infection from biological agents at the workplace into four risk groups [196], whereby moulds fall into risk groups 1 and 2:

- Risk group 1: Biological agents that are unlikely to cause human disease.
- Risk group 2: Biological agents that can cause human disease and might pose a hazard to workers; it is unlikely to spread to the community; effective prophylaxis or treatment is usually available.
- Risk group 3: Biological agents that can cause severe human disease and pose a serious hazard to workers; there may be a risk of spreading to the community, but effective prophylaxis or treatment is usually available.
- Risk group 4: Biological agents that can cause severe human disease and pose a serious hazard to workers; there may be a high risk of spreading to the community; effective prophylaxis or treatment are usually not available (risk group 4 does not include any fungi).

Mould mycoses are opportunistic infections. They require exposed individuals to have reduced immune status. Heat-tolerant mould species in risk group 2 (e. g. *A. fumigatus, A. terreus, A. niger, A. flavus, Emericella nidulans* and mesophilic *Fusarium* sp.) of the classification of moulds into risk groups according to the Technical Rules on Biological Agents (TRBA 460) [195] in the German Biological Agents Act [196] only rarely cause infections in healthy, immunocompetent individuals; however, they can trigger mycosis in individuals whose immune system is incompetent due to disease or other factors [197, 198].

According to KRINKO at the Robert Koch-Institute [11], immunosuppressed individuals can be classified into three risk groups (Table 4). Patients at particular risk include (in descending order of risk): cancer patients, in particular those with underlying hemato-oncological disorders (e. g. leukaemia, lymphoma), acute myeloid leukaemia (AML), acute lymphatic laeukemia (ALL), allogeneic stem cell transplantation, autologous stem cell transplantation, solid organ transplantation, HIV infection, other forms of immunosuppression (e.g. a long-term high-dose treatment with glucocorticoids), aplastic anaemia and cystic fibrosis, among many others [121, 199]. AML is associated with the highest incidence of invasive mould infections (around 12%) and the most mould infections (around 8%). This is followed by ALL at around 4%. Among the procedures, allogeneic hematopoietic stem cell transplantation (allo-HSCT) is associated with an extremely high incidence of mould infections [121].

**Table 4:** Risk groups among immunosuppressed individuals according to the Commission for Hospital Hygiene and Infection Prevention (*Kommission für Krankenhaushygiene und Infektionsprävention*, KRINKO) at the Robert Koch-Institute [11]

**Risk group 1 (moderate immunosuppression/-deficiency)** – Granulocytopenia <0.5 × 10^9^/l (< 500/μl) for up to 10 days (similarly, leukopenia <1 × 10^9^/l; < 1000/μl) – Deficiency of CD4-positive T‑helper cells <250/μl (note: age-appropriate normal values in children); autologous stem cell transplantation up to 3 months following intensive treatment phase *Patients exhibiting more than one feature of immunosuppression/-deficiency listed in risk group 1 come under risk group 2*
**Risk group 2 (severe immunosuppression/-deficiency)** – Granulocytopenia <0.5 × 10^9^/l (< 500/μl) for more than 10 days (similarly, leukopenia <1 × 10^9^/l; < 1000/μl) – Severe aplastic anaemia or macrophage activation syndrome under intensive immunosuppressive treatment – Allogeneic bone marrow or stem cell transplantation up to 6 months following completion of intensive treatment (important: degree of the graft-versus-host disease (GVHD) and continued iatrogenic immunosuppression) – Acute inpatient treatment phase in autologous stem cell transplantation or following solid organ transplantation (up to hospital discharge)
**Risk group 3 (very severe immunosuppression/-deficiency)** – Allogeneic bone marrow transplantation/allogeneic blood stem cell transplantation (PBSCT) in the intensive treatment phase (until engraftment = regeneration of granulopoiesis) – Severe grade III or IV GVHD under intensive immunosuppression *The decision on whether to assign allogeneic stem cell transplantation patients to group 3 is ultimately taken by considering an overview of all findings from the treating oncologists*

Due to the continuously rising proportion of immunosuppressed patients in the population and the ever longer survival rates among this patient group, it is not possible at present to exclude the possibility that mould infections may become a growing health risk factor in this population group [8].

**Fig. 1:**
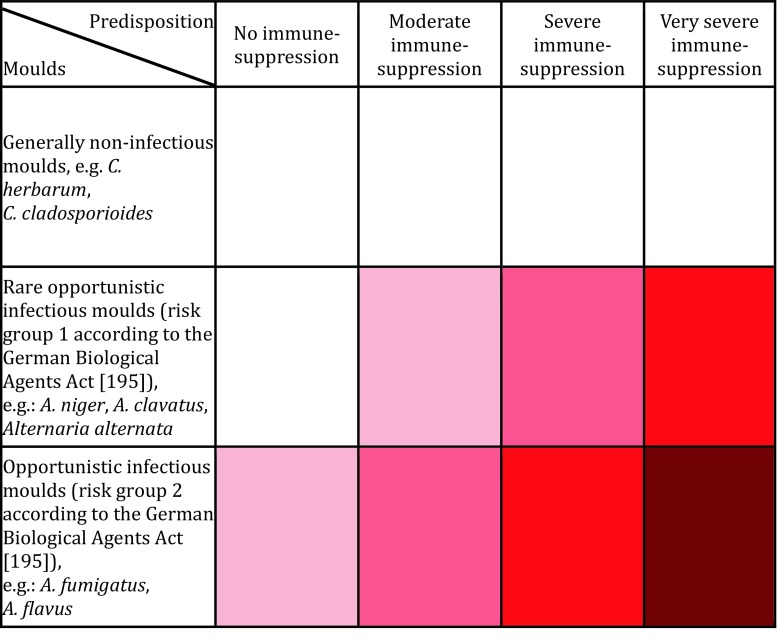
Risk matrix 1: Risk of mould-related infection (the darker the box, the greater the possible health risk)

It is not possible to calculate a numerical risk on the basis of current knowledge. Risk matrix 1 (Fig. 1) shows a semiquantitative risk assessment of the risk of infection due to indoor moulds.

### Risk of sensitization/allergy

In principle, there is a possibility of sensitization and the triggering of a clinically symptomatic allergy also among healthy individuals following inhalation of spores and other mould components (e. g. mycelium). The sensitizing potential of moulds is considered significantly lower [177, 202–204] compared with allergens from, e. g. fur-bearing pets, grass and tree pollen or house dust mites [200, 201]. Recent population- and patient-based [13] studies reveal a comparatively low prevalence of sensitization across Europe of 3–10% measured relative to the total population [12]. It can be said as a general rule that sensitization—also to moulds—is not equivalent to a clinically relevant allergy. It is generally assumed that there are over one million mould species. Around 350 mould species have been listed on www.allergome.org as potentially sensitizing. However, it is not possible to infer how high the total percentage of sensitizing moulds is on the basis of this information. At present, 107 mould proteins from 43 mould species fulfil the WHO/IUIS criteria for classification as an allergen (www.allergen.org). Only a handful of moulds are available as test allergen solutions and typical indoor fungal allergen extracts are largely lacking [38, 205].

From an allergological perspective, an exposure dose-dependence (measured as colony forming units, CFU) does not alone determine the clinical reaction in mould-sensitized patients. Sensitization with the formation of specific IgE antibodies and the triggering of allergic reactions takes place on the protein or peptide-component level. Thus, it is not necessary for whole spores or intact mould mycelium to be present. Allergenicity depends far more on the proteins or peptides which, due to their properties, are allergy-triggering.^2^

Exposure to damp indoor spaces represents a risk factor for developing bronchial asthma in individuals with atopy, rhinoconjunctivitis and rhinosinusitis. In the case of rhinosinusitis associated with mould exposure, the risk for developing bronchial asthma doubles (Odds ratio [OR]: 2.2; confidence interval [CI]: 1.3–3.6) [206]. Young atopic children appear to be at higher risk for developing bronchial asthma in the case of moisture damage or mould exposure in the bedroom or living room [207].

**Fig. 2:**
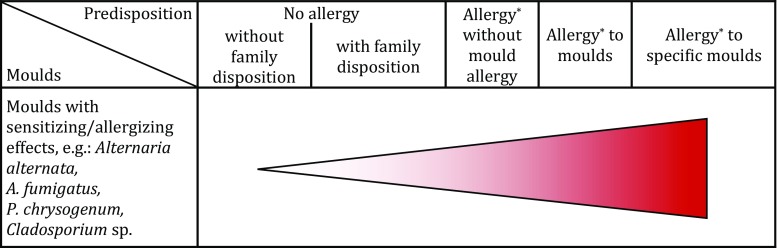
Risk matrix 2: Risk of mould-related sensitization/allergization (the darker the box, the greater the health risk).*Evidence is required of the clinical relevance of sensitization as detected by allergy testing!

It is not possible to calculate a numerical risk value on the basis of current knowledge. Risk matrix 2 (Fig. 2) shows a semiquantitative risk assessment of the risk of sensitization/allergy due to indoor moulds.

### Risk of toxic/irritative effects

Only those moulds that are potentially able to form toxins come into consideration as triggers of intoxication. Whether indoor toxin formation takes place in individual cases depends on environmental and growth conditions and, in this regard, most notably on the substrate [150, 208].

No predisposing factors for mycotoxin intoxication in humans are known. However, predisposition on a target-organ level is conceivable. For example, it is imaginable that pre-existing liver disease (e. g. chronic hepatitis, liver cirrhosis) could represent a predisposition for the hepatotoxic effects of aflatoxin following oral ingestion of this toxin. Whether this also applies to airborne toxin intake remains hitherto unclear [8].

It is not possible to calculate a numerical risk on the basis of current knowledge [43].

It is unclear to date whether persons affected by MMI or chronic bronchitis are particularly sensitive individuals and react even to small doses, or whether sensitized individuals react differently, independent of dose, compared with non-sensitized individuals [157]. Other inflammatory processes in the area of the mucosa of the eyes and respiratory tract, such as infections, atopic mucosal disorders, keratoconjunctivitis sicca and dry nasal mucosa, can be possible predisposing factors for MMI and chronic bronchitis [8].

### Risk of odour effects and mood disorders

In principle, odour effects and/or mood disorders as a result of indoor moisture/mould damage can affect anyone. This does not represent a health risk. Predisposing factors for odour effects may include genetic and hormonal influences, character, context, and adaptation effects [186].

Predisposing factors for mood disorders may be environmental concerns, fears, conditioning, and attributions, as well as numerous diseases [209].

## Diagnostic work-up

### Reason for seeking medical advice

Patients generally seek medical advice in conjunction with mould exposure for the following reasons [105, 162]:

1. Patients experience health problems, the circumstances of which suggest an environment-related link to moisture damage and/or mould exposure.
2. Patients have mood disorders and nonspecific symptoms that are in clear temporal relationship to certain environmental/ambient conditions or activities.
3. Patients are concerned about possible mould exposure.
4. Measurements are already available.
5. Medical support is sought in rental and construction disputes.

### Diagnostic work-up: general procedure, medical history, physical examination and clinical chemical and instrument-based tests

Medical history [210] and physical examination are the basic elements of any medical diagnostic work-up. On the basis of these, further special investigations are performed within the relevant medical specialty depending on the diagnostic question and differential diagnostics. In addition, in environmental and occupational medicine, investigations to internal exposure (human biomonitoring in the form of exposure and/or effect monitoring) and/or external exposure (home/site visit, environmental monitoring) are always performed if possible and where indicated.

### Medical history

Medical history taking should involve a holistic approach that is not confined to environmental exposure and aspects of physical disease alone, but one that also takes the psychosocial dimension equally into account. This approach, which is particularly necessary in view of the patient’s high expectations of the physician, should be explained to the patient. Giving equal priority to psychological and social aspects rarely produces difficulties in the consultation setting once the approach has been explained to the patient.

In addition to the general and differential diagnostic history, the following elements should be considered during medical history taking in the case of suspected health disorders due to mould:

- history of exposure in the home,
- history of exposure in the workplace,
- history of exposure during leisure time,
- history of infections, including predisposing factors,
- history of allergies, including predisposing factors,
- history regarding irritant toxic effects,
- history regarding odour effects,
- history regarding mood disorders.

#### Physical examination

A complete, or at least symptom-oriented, physical examination always forms an integral part of medical history taking. The method of physical examination is based on inspection, palpation, percussion, auscultation and functional testing.

The target organs noted in the medical history should take priority in the examination. Particular attention should be paid to the mucosa of the eyes and, as far as possible, upper airways, as well as to the skin, since the nonspecific symptoms that patients often complain of relate to these organs in particular [22, 211]. As a basic rule, the physical examination should be performed in a structured and standardized manner and should be adequately documented. A variety of clinical finding forms are available to this end.

### Markers of mould exposure

#### Environmental monitoring

As a rule, there is no medical indication to determine mould indoors, in building materials or on fixtures.

From a medical perspective, a visual inspection of mould infestation is sufficient to initiate medically justified measures. The greatest relevance is attributed to site visits, which are ideally performed by a physician and experts with structural expertise.

In the case of visible mould infestation, increased moisture levels in materials or structural abnormalities (moisture or water damage), the identification and quantification of indoor mould is not indicated from a diagnostic and therapeutic perspective [212].

The differential diagnosis always takes priority when assessing the health effects of mould exposure. Since the effect of mould depends primarily on the disposition of the affected individual, any delay caused through mould determination in taking steps may put persons requiring particular protection from mould at increased risk. Risk groups warranting particular protection include:

- immunosuppressed individuals according to the three risk groups defined by the KRINKO at the Robert Koch-Institute [11],
- individuals with cystic fibrosis (mucoviscidosis),
- individuals with bronchial asthma.

In the above-mentioned patient groups, tests on the basis of relevant suspicion are seldom medically indicated to assess risk purely for preventive purposes.

Gabrio et al. (2015) recently presented a summary of test methods available to ascertain mould exposure in indoor mould infestation, e. g. due to moisture damage [213]. Their summary is designed to provide not only treating physicians, but also environmental mycologists, indoor diagnosticians, craftsmen, architects and building experts responsible for ordering or evaluating measurements, with sound knowledge about useful (or redundant) applications and the reliability of the various measuring and test methods, thereby making a solid basis available for the commissioning or assessment of relevant tests. The reader is referred to the pertinent literature for a more in-depth discussion of this topic [2, 4, 214, 215].

### Clinical diagnostics

#### 1. Allergy diagnostic work-up

The diagnostic work-up here does not differ from that in other allergic diseases. A stepwise approach is taken that considers individual factors according to the classic step-by-step model: medical history/physical findings/clinical investigation—skin tests—serum analysis or complementary *in vitro* methods—provocation [89, 216].

Allergic disorders due to mould allergens can essentially manifest as conjunctivitis, rhinitis, rhinosinusitis, allergic bronchial asthma, urticaria, HP and ABPA. As a result, the differential diagnosis based on medical history and clinical laboratory *in vitro*/*in vivo* testing is of central importance. In individual cases, the allergic reaction needs to be confirmed and the allergy trigger identified. There is a wide variety of *in vitro* tests to measure parameters of the cellular and humoral allergic reaction on different levels. However, the repertoire of commercially available mould allergen extracts is limited and primarily covers typical species found in outdoor air.

Particularly in *in vitro* testing, it is important to bear in mind that increased mould-specific IgE levels, for instance, can indicate sensitization to mould allergens, but do not equate to allergic disease. A correct interpretation of results is only ever possible in conjunction with the medical history, clinical picture and/or the results of organ-specific provocation tests. Given the possibilities for exposure (ubiquitous outdoor exposure, indoor exposure and occupational exposure), evidence of positive sensitization to mould needs to be regarded critically in the assessment of causality. It is extremely rare in routine allergology/environmental medicine for a causal link to be reliably confirmed between indoor mould exposure and an associated specific sensitization and disease (rhinitis, conjunctivitis, asthma) [8].

The following conditions need to be met in order for a mould allergy to be diagnosed [217]:

- A pathogenic mould antigen is present in the environment.
- There is an unequivocal temporal relationship between allergic symptoms and exposure to the mould allergen.
- Atopic predisposition is present.
- There is evidence of specific IgE formation to mould antigens.
- Measures to avoid mould allergens exhibit clear clinical effects.

In principle, the same recommendations and guidelines apply to the diagnostics of mould allergy as to other allergen sources that cause immediate-type allergies [218].

##### 1.1 Serological investigations.

Serological *in vitro* tests include specific IgE antibody determination in the case of IgE-mediated disease, or specific IgG antibody determination in the case of HP. Although the identification of elevated specific antibodies is a clear indication of sensitization, this does not equate to clinical relevance; having said that, the predictive value for clinical relevance increases according the degree of sensitization [219].

##### 1.2 Identification of mould-specific IgE antibodies.

The identification of allergen-specific IgE indicates specific sensitization, but not necessarily disease; results can only be correctly interpreted in conjunction with medical history, clinic picture and the results of organ-specific provocation tests. Positive reactions caused by cross-sensitivity are of only partial clinical relevance.

A quantitative comparison of results from different test systems is challenging (call for international standards).

A call needs to be made for the improvement of reagent quality by standardizing the allergens and defining minimum requirements for the allergen-carrier material (determining diagnostic efficacy).

Extracts of indoor-relevant moulds should also be commercially available in adequate quality.

The spectrum of available single allergens from the relevant moulds needs to be expanded.

##### 1.3 Mould-specific IgG determination.

Only in the case of suspected ABPA (type I and III allergy) or HP (type III and IV allergy) can mould-specific IgG antibody determination make a helpful contribution to diagnosis and, as such, be recommended [101, 107].

In ABPA, a significant increase is seen not only in total IgE and specific IgE against *A. fumigatus* (see above), but also in specific IgG against *A. fumigatus*. The latter is markedly elevated compared with patients allergically sensitized to *A. fumigatus* and is therefore recommended in the differential diagnosis of ABPA.

##### 1.4 Cytokines and eosinophil cationic protein (ECP).

There is no special indication for these nonspecific markers of eosinophil activation and recruitment in the identification of mould allergy.

##### 1.5 Immune complex analysis.

The analysis of immune complexes is confined to particular disorders in the realm of type III allergic reactions, such as HP, and has no place in the diagnostics of mould exposure beyond this (see mould-specific IgG determination above).

##### 1.6 Galactomannan in serum.

The detection of serum galactomannan for diagnostic purposes is only indicated in invasive aspergillosis [220].

##### 1.7 β-1,3-D-glucan in serum.

The detection of (1→3)-ß-D-glucan in serum is technically challenging and could be helpful in the diagnostic work-up of invasive mycosis. Its application is not indicated in conjunction with indoor mould [221].

##### 1.8. Mycotoxins in serum.

Current analytical possibilities do not permit the reliable determination or evaluation of indoor mycotoxin exposure. The determination of mycotoxins in blood, serum or urine is of no relevance in practical medicine and must remain confined to scientific investigations for the time being.

#### 2. Cellular assays

Rare indications for tests with the “basophil granulocyte” target cell include samples with extremely low total IgE and failed specific serological IgE detection in the case of suspected sensitization or exotic allergens.

##### 2.1 The basophil degranulation test and histamine release.

The histamine release test (HRT) is not helpful in the diagnostics of mould allergy.

##### 2.2 The basophil activation test by flow cytometry (Flow CAST).

This test is beneficial in the case of inhalant allergens, particularly when skin testing and specific IgE measurements are not possible. Rare indications include samples with low total IgE, failed serological specific IgE detection and possibly negative skin tests in suspected sensitization or exotic allergens.

##### 2.3 Determination of other effector-cell mediators (leukotriene release test, cellular antigen stimulation test [CAST]).

Testing positive to an allergen, which represents an indirect identification of sensitization, is only indicative of a clinically relevant allergy in conjunction with a positive medical history and/or positive provocation tests. This test is also complex to perform and not suited to routine diagnostics.

##### 2.4 Lymphocyte transformation test.

Since mould allergens do not cause type IV sensitization, lymphocyte transformation testing for mould is not indicated as a diagnostic method [14].

#### 3. Provocation tests

In cases where medical history, physical examination and serology fail to unequivocally establish the diagnosis of a mould allergy, provocation testing may be indicated if this will significantly impact treatment, prevention and/or compensation [222]. Organ-specific provocation testing is aimed at confirming the clinical relevance of existing sensitizations or supposedly observed symptoms.

At present, only a few commercial mould allergen test extracts are available from a handful of manufacturers. As shown in investigations published by Kespohl et al. [223] in 2013 using detailed biochemical and immunological analysis, mould allergen extracts exhibit high variability in terms of allergen composition, and preparations of a fungal species are not comparable between different manufacturers. Skin test extracts of the outdoor mould *Alternaria* are an exception here.

##### 3.1 Skin testing.

After medical history, skin tests (ST) form the basis of the allergy diagnostic work-up and are fast and relatively cost-effective to perform. As a general rule, they are sufficiently meaningful and are associated with a low complication rate. ST should be performed according to the relevant German or European position papers [224].

A distinction is made in skin testing between epicutaneous (patch, friction) and cutaneous (scratch, skin prick and intracutaneous) tests. The allergen concentration in solutions used for intracutaneous testing is usually 100- to 1000-fold lower compared with skin prick test solutions. However, since intracutaneous test solutions have not been commercially available since June 2015, this diagnostic method to detect mould sensitization no longer applies.

##### 3.2 Nasal provocation testing.

The nasal provocation test (NPT) makes it possible to reproduce an allergic reaction at the manifesting organ under standardized conditions and is considered a simple and safe method with high specificity and sensitivity [224–227]. It is recommended that tests be performed and evaluated according to standards set out in the German Society for Allergology and Clinical Immunology (*Deutsche Gesellschaft für Allergologie und klinische Immunologie*, DGAKI) guideline [228].

Inhalation allergies to mould spores generally cause persistent respiratory tract symptoms; this can make it challenging to establish an unequivocal relationship to the medical history. In this context, NPT is able to confirm or exclude a suspected diagnosis of an allergic reaction of the respiratory tract. NPT is also indicated in cases where skin testing is contraindicated or local allergic rhinitis is suspected, and to monitor the course of treatments such as allergen specific immunotherapy (SIT).

##### 3.3 Conjunctival provocation testing.

The conjunctival provocation test (CPT) should only be performed when the patient is free of symptoms; standardized skin prick test solutions (1:10 dilution, possibly higher) are generally used [229].

A CPT may be indicated if

- symptoms are predominantly conjunctival,
- an NPT for nasal symptoms is not possible due to contraindications or recent endonasal surgery.

##### 3.4 Bronchial provocation testing.

A bronchial provocation test (BPT) may be indicated if it is not possible to establish the diagnosis on the basis of a combination of exposure tests and less invasive diagnostic tools, such as medical history and skin testing. Medical history is generally not helpful, particularly in perennially occurring indoor moulds. Optionally, there is an indication to confirm diagnosis prior to hyposensitization, as well as in cases where a court-admissible expert opinion on a link to a particular instance of exposure is required [222]. As with other inhalant allergens, the degree of sensitization can be taken into account as a guide. As such, the BPT plays an important role in suspected allergic perennial asthma due to indoor moulds. Allergen selection should be guided by the spectrum of sensitization. The evidence for provocation testing in the event of failure to detect sensitization is insufficient, meaning that no recommendation can be made in this regard. The spectrum of commercial extracts available for provocation testing is progressively narrowing. The test must be performed according to the relevant guidelines. When assessing allergen provocation tests, one should expect false-positive as well as false-negative reactions. It is generally problematic to make any statements on sensitivity and specificity in the absence of a clinically relevant gold standard, a situation made more challenging in the case of moulds due largely to the lack of investigations on the quality of test extracts. Recent Finnish studies on occupational mould exposure show that provocation testing with commercial mould extracts may be significantly more sensitive compared with the detection of sensitization [230]. These data require validation. It is therefore challenging to assess provocation reactions in mould provocation tests, partly since often isolated delayed reactions are described [230].

#### 4. Diagnostic work-up for infections

The reader is referred to the relevant guideline for details on the procedure in mould infections (systemic mycoses).

#### 5. Toxicological diagnostics

There are currently no practicable and validated test methods that could be applied in clinical diagnostic practice.

#### 6. Unconventional diagnostic methods

Due to a lack of evidence, unconventional (or unconventionally used) diagnostic methods [231–233] are not advised.

## Treatment

Even if no causal link can be established between symptoms/findings/disorders and the occurrence of indoor mould/dampness, the first “therapeutic” measure to be undertaken from a preventive and hygienic perspective in the case of dampness/mould damage is prompt appropriate and professional remediation; moreover, in the case of severe clinical pictures associated with high risk (immune suppression according to KRINKO criteria [11], cystic fibrosis [mucoviscidosis], asthma), immediate minimization of exposure needs to be achieved.

### General drug treatment

In principle, topical and/or systemic treatment is indicated in mould allergy depending on the organ-specific manifestation of the allergic disorder.

The reader is referred to the relevant guidelines for more details on (organ-specific) drug treatment of allergies.

### Specific immunotherapy (hyposensitization)

Specific immunotherapy (SIT) using mould extracts should be applied as early in the disease course as possible, particularly if drug treatment and avoidance have previously failed to stabilize symptoms [234]. The relevant mould allergens need to be unequivocally confirmed at diagnosis as the trigger of allergic symptoms. The prerequisite for SIT is evidence of clinically relevant allergen-specific IgE sensitization. The combination of different test methods, together with medical history, provides an adequate basis for SIT. Hyposensitization presupposes a confirmed diagnosis. In this regard, the reader is referred to the current guideline [235].

According to current data, only a handful of studies support the efficacy of SCIT in the outdoor-relevant moulds *Alternaria alternata* and *Cladosporium herbarum* [236, 237].

There is insufficient scientific evidence to date to support the efficacy of sublingual immunotherapy (SLIT) in terms of hyposensitization to indoor-relevant moulds.

### Exposure avoidance

As with all allergic diseases, exposure avoidance (allergen avoidance) takes priority. Nevertheless, prompt medication is required in order that a symptom-free period is not followed by full-blown allergic disease. It is of paramount importance to eliminate the causes of the dampness creating a basis for indoor mould growth. The AWMF mould guideline [1] provides recommendations for indoors, outdoor air and foods (recommendations without evidence).

### Unconventional treatment methods

As with all medical procedures, unconventional treatment procedures [231–233] need to be tested and evaluated according to current scientific knowledge and will only be reimbursed by health insurers if the therapeutic benefit is proven.

## Remediation of living areas (buildings) affected by dampness and mould growth

Proper remediation of dampness/mould damage includes the elimination of structural cause(s), the drying out and removal of all mould-infested materials as well as subsequent fine cleaning. Details of these procedures do not form part of this guideline. More detailed information can be found in the relevant mould guidelines [2, 3, 5], as well as the revised version of the UBA guideline (due to be published 2017).

## Social status and dampness/mould infestation

Statistical surveys show that dampness/mould damage is more frequently reported in homes of individuals with low social status compared with the general population (e. g. German Federal Statistical Office, 2006). This gives rise to particular problems for low-social-status individuals in terms of the likelihood of dampness/mould damage and its remediation [1].

## Prevention

It is important, as a first step, to provide susceptible and immunosuppressed patients with information on the risks associated with indoor mould exposure and preventive measures [238, 239], possibly supplemented by home visits to inspect for *Aspergillus fumigatus* and *Aspergillus flavus* (only rarely found indoors) [240].

Prevention and exposure avoidance are paramount in all cases of health-related disorders associated with exposure to environmental factors. This applies in particular to moulds. To ensure allergy prevention, it is essential to avoid an indoor climate that promotes mould growth (high air humidity, lack of ventilation) [241].

Further information can be found in the UBA guideline on mould [2], as well as the revised version of the UBA guideline (due to be published 2017).

### Consensus procedure.

Guideline Commission, the German Society of Hygiene, Environmental Medicine and Preventive Medicine (GHUP) (*Gesellschaft für Hygiene, Umweltmedizin und Präventivmedizin*, GHUP)

### Facilitator.

Prof. Dr. Gerhard A. Wiesmüller, Cologne, Germany

## Footnotes

1 Sometimes also referred to as mucous membrane irritation syndrome (MMIS).

2 Definition of the term “allergen”: Allergens are antigens which cause allergy. Most allergens reacting with IgE and IgG antibody are proteins, often with carbohydrate side chains, but in certain circumstances pure carbohydrates have been postulated to be allergens. In rare instances, low molecular weight chemicals, e.g. isocyanates and anhydrides acting as haptens, are still referred to as allergens for IgE antibodies (WAO/EAACI Allergy Definitions, http://www.worldallergy.org/professional/allergic_diseases_center/nomenclature/english.php).
